# The founding charter of the Genomic Observatories Network

**DOI:** 10.1186/2047-217X-3-2

**Published:** 2014-03-07

**Authors:** Neil Davies, Dawn Field, Linda Amaral-Zettler, Melody S Clark, John Deck, Alexei Drummond, Daniel P Faith, Jonathan Geller, Jack Gilbert, Frank Oliver Glöckner, Penny R Hirsch, Jo-Ann Leong, Chris Meyer, Matthias Obst, Serge Planes, Chris Scholin, Alfried P Vogler, Ruth D Gates, Rob Toonen, Véronique Berteaux-Lecellier, Michèle Barbier, Katherine Barker, Stefan Bertilsson, Mesude Bicak, Matthew J Bietz, Jason Bobe, Levente Bodrossy, Angel Borja, Jonathan Coddington, Jed Fuhrman, Gunnar Gerdts, Rosemary Gillespie, Kelly Goodwin, Paul C Hanson, Jean-Marc Hero, David Hoekman, Janet Jansson, Christian Jeanthon, Rebecca Kao, Anna Klindworth, Rob Knight, Renzo Kottmann, Michelle S Koo, Georgios Kotoulas, Andrew J Lowe, Viggó Thór Marteinsson, Folker Meyer, Norman Morrison, David D Myrold, Evangelos Pafilis, Stephanie Parker, John Jacob Parnell, Paraskevi N Polymenakou, Sujeevan Ratnasingham, George K Roderick, Naiara Rodriguez-Ezpeleta, Karsten Schonrogge, Nathalie Simon, Nathalie J Valette-Silver, Yuri P Springer, Graham N Stone, Steve Stones-Havas, Susanna-Assunta Sansone, Kate M Thibault, Patricia Wecker, Antje Wichels, John C Wooley, Tetsukazu Yahara, Adriana Zingone

**Affiliations:** 1 Gump South Pacific Research Station, University of California Berkeley, BP 244 98728 Moorea, French Polynesia; 2 Biodiversity Institute, Department of Zoology, University of Oxford, The Tinbergen Building, South Parks Road, Oxford OX1 3PS, UK; 3 Oxford e-Research Centre, University of Oxford, 7 Keble Road, Oxford OX1 3QG, UK; 4 Centre for Ecology & Hydrology, Maclean Building, Benson Lane, Crowmarsh Gifford, Wallingford, Oxfordshire OX10 8BB, UK; 5 The Josephine Bay Paul Center for Comparative Molecular Biology and Evolution Marine Biological Laboratory, Woods Hole, Massachusetts, MA 02543, USA; 6 British Antarctic Survey, Natural Environment Research Council, High Cross, Madingley Road, Cambridge CB3 0ET, UK; 7 Berkeley Natural History Museums, 1007 Valley Life Sciences, University of California, Berkeley, CA 94720, USA; 8 Department of Computer Science, University of Auckland, Auckland 1142, New Zealand; 9 Allan Wilson Center for Molecular Ecology and Evolution, University of Auckland, Auckland, New Zealand; 10 The Australian Museum, 6 College St., Sydney, NSW 2010, Australia; 11 Moss Landing Marine Laboratories, California State University, 8272 Moss Landing Road, Moss Landing, CA 95039, USA; 12 Institute for Genomic and Systems Biology, Argonne National Laboratory, 9700 South Cass Avenue, Argonne, IL 60439, USA; 13 Department of Ecology and Evolution, University of Chicago, 5640 South Ellis Avenue, Chicago, IL 60637, USA; 14 Microbial Genomics and Bioinformatics Research Group, Max Planck Institute for Marine Microbiology, D-28359 Bremen, Germany; 15 Jacobs University Bremen, D-28759 Bremen, Germany; 16 Rothamsted Research, Harpenden, Herts AL5 2JQ, UK; 17 Hawaii Institute of Marine Biology, School of Ocean & Earth Science & Technology, University of Hawaii at Manoa, PO Box 1346, Kaneohe, HI 96744, USA; 18 Department of Invertebrate Zoology, National Museum of Natural History, Smithsonian Institution, PO Box 37012, MRC-163, Washington, DC 20013, USA; 19 Department of Biological and Environmental Sciences, University of Gothenburg, Box 463 SE-405 30 Gothenburg, Sweden; 20 USR 3278 CNRS - EPHE Centre de Recherche Insulaire et Observatoire de l’Environnement (CRIOBE), BP 1013 - 98 729 Papetoai, Moorea, French Polynesia; 21 Monterey Bay Aquarium Research Institute, Moss Landing, CA 95039, USA; 22 Department of Life Sciences, Imperial College London, London SW7 2AZ, UK; 23 Department of Life Sciences, Natural History Museum, London SW7 5BD, UK; 24 Independent 316 Scientific Adviser, 2 rue de la Tour de Magnan, 06 000 Nice, France; 25 National Museum of Natural History, Smithsonian Institution, PO Box 37012, 318 MRC 106, Washington, DC 20013, USA; 26 Department of Ecology and Genetics and Science for Life Laboratory, Uppsala University, Norbyv. 18D, SE-75236 Uppsala, Sweden; 27 Department of Informatics, University of California, Irvine, Irvine, CA 92697, USA; 28 PersonalGenomes.org, Boston, MA 02215, USA; 29 CSIRO Marine and Atmospheric Research and Wealth from Oceans National Research Flagship, Hobart, Tasmania, Australia; 30 AZTI-Tecnalia, Marine Research Division, Herrera Kaia, Portualdea s/n, 20110 Pasaia, Spain; 31 Department of Biological Sciences, University of Southern California, Los Angeles, CA 90089-0371, USA; 32 Alfred Wegener Institute for Polar and Marine Research, Biologische Anstalt Helgoland, 27498 Helgoland, Germany; 33 Essig Museum of Entomology, University of California, Berkeley, CA 94720, USA; 34 National Oceanic and Atmospheric Administration, AOML stationed in La Jolla, San Diego, CA 92037, USA; 35 Center for Limnology, University of Wisconsin, MadisonWI 53706, USA; 36 Environmental Futures Research Institute, Griffith University, Gold Coast, Queensland 4222, Australia; 37 National Ecological Observatory Network, Boulder, CO 80301, USA; 38 Earth Sciences Division, Lawrence Berkeley National Laboratory, Berkeley, CA 94720, USA; 39 Station Biologique de Roscoff, CNRS - UPMC, Place Georges Teissier, CS 90074, 29688 Roscoff Cedex, France; 40 Denver Botanic Gardens, 909 York Street, Denver, CO 80206, USA; 41 Howard Hughes Medical Institute, Chevy Chase, Maryland, USA; 42 Departments of Chemistry & Biochemistry and Computer Science, and BioFrontiers Institute, University of Colorado at Boulder, 3415 Colorado Ave, Boulder, CO 80309, USA; 43 Museum of Vertebrate Zoology, University of California, Berkeley, CA 94720-3160, USA; 44 Institute of Marine Biology, Biotechnology and Aquaculture (IMBBC), Hellenic Centre for Marine Research (HCMR), Heraklion, Greece; 45 Terrestrial Ecosystem Research Network and Environment Institute, University of Adelaide, Adelaide SA5005, Australia; 46 Food Safety, Environment & Genetics, Matis ldt. Vinlandsleid 12, 113 Reykjavik, Iceland; 47 School of Computer Science, The University of Manchester, Oxford Road, Manchester M13 9PL, UK; 48 Department of Crop and Soil Science, Oregon State University, Corvallis, OR 97331, USA; 49 Biodiversity Institute of Ontario, University of Guelph, Guelph, Ontario N1G 2W1, Canada; 50 Environmental Science Policy and Management, University of California, Berkeley, CA 94720-3114, USA; 51 AZTI-Tecnalia, Marine Research Division, Txatxarramendi ugartea z/g, Sukarrieta 48395Bizkaia, Spain; 52 National Oceanic and Atmospheric Administration, Office of Exploration and Research, Silver Spring, Maryland 20910, USA; 53 Institute of Evolutionary Biology, University of Edinburgh, The King’s Buildings, Edinburgh EH93JT, UK; 54 Biomatters Ltd., Auckland 1010, New Zealand; 55 Center for Research on BioSystems, University of California, San Diego, CA 92093-5004, USA; 56 Center for Asian Conservation Ecology, Kyushu University, 6-10-1, Fukuoka 812-8581, Japan; 57 Ecology and Evolution of Plankton Laboratory, Stazione Zoologica Anton Dohrn, Villa Comunale, 80121 Naples, Italy

**Keywords:** Biodiversity, Genomics, Biocode, Earth observations

## Abstract

The co-authors of this paper hereby state their intention to work together to launch the *Genomic Observatories Network* (GOs Network) for which this document will serve as its *Founding Charter*. We define a Genomic Observatory as an ecosystem and/or site subject to long-term scientific research, including (but not limited to) the sustained study of genomic biodiversity from single-celled microbes to multicellular organisms.

An international group of 64 scientists first published the call for a global network of Genomic Observatories in January 2012. The vision for such a network was expanded in a subsequent paper and developed over a series of meetings in Bremen (Germany), Shenzhen (China), Moorea (French Polynesia), Oxford (UK), Pacific Grove (California, USA), Washington (DC, USA), and London (UK). While this community-building process continues, here we express our mutual intent to establish the GOs Network formally, and to describe our shared vision for its future. The views expressed here are ours alone as individual scientists, and do not necessarily represent those of the institutions with which we are affiliated.

## Background

Key outcomes of 21^st^ century science include an Earth with its essential life support systems intact, and a planet where human society has achieved sustainable development. Achieving these challenges, however, requires a greatly improved understanding of human interactions with the natural environment. Towards that end, the GOs Network aims to observe DNA sequences -- the biocode -- across the principal levels of biological organization (cell, organism, ecosystem) up to the planetary genome [1,2]. Our approach is to apply genomic technologies to study the flux of genetic variation within these nested scales of biological function. We will inventory genomic biodiversity and map its distribution over time and space. To address the processes that generate and maintain this diversity, we will link the genomic information to physico-chemical, ecological, and socioeconomic data. We will work with the broader scientific community to build models of how genomic biodiversity contributes to ecosystem services, evolutionary potential, and ecological resilience.

## Main text

### What is a Genomic Observatory?

Some of the terms we use here require definition (See Table 1), since not everyone in the community uses them in the same way. Helping to solidify a shared terminology in such a fast moving field is one contribution of the GOs Network. Our intention here, however, is merely to clarify what we mean in the limited context of this document. Only time will tell whether these terms, or our definitions of them, become broadly accepted. The definitions are deliberately concise and in some cases require fuller explanation, which we plan to provide in a future publication.

**Table 1:** Definitions

**Biocode**	The totality of DNA sequences in a given *unit of biological organization,* such as a: *cell* (e.g., the Yeast Biocode includes both its nuclear and mitochondrial genomes); *organism* (e.g., the Human Biocode includes both the Human Genome and the Human Microbiome); *ecosystem* (e.g., the Moorea Biocode includes all the genomes on the island); *planet* (e.g., the Earth Biocode includes all the genomes on the planet)
**Biocoding the earth**	The aspirational target of sequencing every genome on the planet. While a theoretical goal that is clearly unattainable in practice, strategic genome sequencing (e.g., as proposed by the Global Genome Initiative http://www.mnh.si.edu/ggi/) can cover the major variation found among genomes on Earth
**Planetary genome**	A special case of the biocode: the sum of all genomes that exist on Earth at a given time. (N.B. (nota bene): the existence of a planetary genome neither implies that natural selection acts at this level, nor that the phenotype of the planetary genome is adapted for its preservation and propagation)
**Genomic biodiversity**	The genetic variation found among genomes
**Biodiversity genomics**	The field of scientific study that maps genomic biodiversity over space and time, investigates the functional consequences of this variation, and seeks to explain how it is generated and maintained
**Ecosystem**	A biological community of interacting organisms in their physical and chemical environment
**Genomic Observatory**	An *ecosystem* and/or site subject to long-term scientific research, including (but not limited to) the sustained study of genomic biodiversity from single-celled microbes to multicellular organisms
**Genomic Observatories (GOs) Network**	A network (i) of *ecosystems* and *sites*, which are often already part of existing scientific networks, (ii) of *researchers*, who are intensively studying one or more GOs, and (iii) of *institutions*, *infrastructures*, and *initiatives*, whose work aligns with the GOs Network’s mission
**Future ‘omics** (futuromics)	The preservation of biological samples for eventual study of their nucleotide and protein sequences through the techniques of genomics, transcriptomics, proteomics, metabolomics, and other ‘omics’ analyses

Most importantly, we define a ‘Genomic Observatory’ as an ecosystem, and/or a site within an ecosystem, that is the subject of long-term scientific research, including (but not limited to) the sustained study of genomic biodiversity. An *observatory* is the institutionalized act of observing, and so for a given ecosystem (and/or site) to be recognized as a Genomic Observatory, one or more institutions (e.g., field station, marine laboratory, museum, university, etc.) should express, and preferably have demonstrated, their long-term commitment to the scientific study of that system’s genomic biodiversity. One important feature of this definition is that it allows the establishment of new genomic observatories, while recognizing the value of those that have existing time-series data already.

### Mission and vision

The mission of the GOs Network is to work towards Biocoding the Earth; integrating DNA data into Earth observing systems and eventually building a global Genomic Observatory within the Global Earth Observation System of Systems (GEOSS) [3].

The vision of the GOs Network is to:

• Advance the science of biodiversity genomics through a global network of premier research organizations generating well-contextualized genomic biodiversity observations compliant with global data standards.

• Coordinate a set of long-term DNA-centric research programs (actions) at local, regional, and global scales that help develop and implement common standards and best practices for quantifying genomic biodiversity and mapping biotic interactions over time.

• Partner with natural history museums, repositories, and bio-banks, (e.g., members of the Global Genome Biodiversity Network (GGBN) [4]) to preserve well-contextualized samples (environmental samples and organismal specimens) for future ‘omics analysis, including whole genomes and metagenomes.

• Work with the broader scientific community to develop predictive models of biodiversity, ecosystem services and evolutionary potential [5] - especially with respect to global change.

• Provide training, technical assistance, resources, and best practice guides as a learning platform for individuals and organizations wishing to carry out genomic observations at genomic observatories and beyond.

### Governance and membership

The GOs Network is a collaboration of the Genomic Standards Consortium (GSC) and the Group on Earth Observations (GEO) through its Biodiversity Observation Network (GEO BON) [6]. The GSC is incubating the GOs Network, and GEO BON has listed it as a key deliverable. Through this pathway, the GOs Network will also contribute to the new Future Earth program [7] for global sustainability research, thus promoting links to other scientific disciplines and helping forge partnerships with policy makers and other stakeholders.

Working under GEO reflects our mission of integrating genomic data into GEOSS, while working under GSC ensures that these data are fit for that purpose. Initially, a GOs Network Board, including representatives of key stakeholders, will administer the GOs Network and set its strategic objectives (this charter). One of the first tasks of the Board is to define criteria for membership of the GOs Network and to put in place a governance mechanism and operating procedures. All co-authors of this article are considered “founding individual members” of the GOs Network.

### Research coordination activities

The GOs Network will focus on coordinating activities, such as (1) Organizing an annual meeting (GOs Network Conference) involving all network members and other interested parties worldwide; (2) Co-organizing regional and thematic meetings (GOs Network Workshops) in collaboration with partner organizations; (3) Maintaining a online registry of Genomic Observatories; (4) Participating in standards development efforts, for example, through the *Genomic Biodiversity Working Group* (GBWG) - a collaboration of the GSC and the Biodiversity Information Standards organization (TDWG.org); and (5) contributing to GEO and GEOSS, particularly through the cross-cutting Working Group 1 (Genetics) of GEO BON.

### Actions

The GOs Network will support actions aiming to build a coordinated and well-contextualized set of genomic biodiversity observations and archived vouchers (specimens and environmental samples). In particular, the GOs Network aims to provide a roadmap for ecosystem-based Genomic Biodiversity Assessment Reports as one of its contributions to GEO BON. These reports might start with a simple checklist of species, building up to a DNA barcode library and eventually to metagenomic inventories. The former will contribute towards the International Barcode of Life (IBOL) initiative and work towards the latter is already underway through the GOs Network’s first action, *Ocean Sampling Day* (OSD) [8], an initiative of the EU FP7 Project Micro B3 to carry out coordinated sampling of marine microbial communities on June 21, 2014. The GOs Network will help expand the scope of OSD to include new geographies, sampling approaches, taxa and environments, and to maintain this action beyond 2014. OSD represents the GOs Network’s first attempt to aggregate participating sites into a global genomic observatory and to begin functioning as a distributed major research infrastructure. The GOs Network will build on OSD to support development of Marine Biodiversity Observation Networks [9], particularly through the coordinated actions of leading marine Genomic Observatories.

### Biocode commons

The GOs Network intends to adopt the Biocode Commons as its informatics stack - a primary forum for sharing tools that support genomic observations from collection through analysis and publication, bringing together developers, scientists and standards (see: http://biocodecommons.org/). Eventually, the Biocode Commons might aim to provide a one-stop shop for genomic biodiversity data and scientific workflows from genomic observatories. We believe that wherever feasible these data should be fully compliant with global data standards, machine-readable, *and* (while respecting legitimate concerns for privacy and for the protection of endangered species) accessible without restriction to the scientific community [10].

## Conclusions

Genomic biodiversity represents the foundational data layer for biological research. The Genomic Observatories (GOs) Network brings together premier research sites to develop ‘systems-based’ approaches that integrate genomics into ecological, evolutionary, and socio-environmental studies. This paper outlines the network’s mission and seeks broad community participation in this collaborative effort.

## Abbreviations

GEO: Group on Earth Observations; GEO BON: Group on Earth Observation Biodiversity Observation Network; GEOSS: Global Earth Observation System of Systems; GGI: Global Genome Initiative; GGBN: Global Genome Biodiversity Network; GSC: Genomic Standards Consortium; IBOL: International Barcode of Life; OSD: Ocean Sampling Day.

## Competing interests

The authors declare that they have no competing interests.

## Authors’ contributions

ND and DF drafted the original text with detailed input from GOs Steering Committee members (LAZ, MC, JD, AD, DPF, JG, JG, FOG, PH, JL, CM, MO, SP, CS, APV, VBL, RG, and RT) and broad consultation with all the co-authors. All authors read and approved the final manuscript.

## GOs-COS

Genomic Observatories Community of Scientists. http://genomicobservatories.org/, Oxford, England, UK; info@genomicobservatories.org.
